# Mechanism of RNA modification N6-methyladenosine in human cancer

**DOI:** 10.1186/s12943-020-01216-3

**Published:** 2020-06-08

**Authors:** Zijian Zhou, Jiancheng Lv, Hao Yu, Jie Han, Xiao Yang, Dexiang Feng, Qikai Wu, Baorui Yuan, Qiang Lu, Haiwei Yang

**Affiliations:** Department of Urology, The First Affiliated Hospital of Nanjing Medical University, Nanjing, 210029 PR China

**Keywords:** N6-methyladenosine, RNA methylation, Cancer

## Abstract

Since the breakthrough discoveries of DNA and histone modifications, the field of RNA modifications has gained increasing interest in the scientific community. The discovery of N6-methyladenosine (m6A), a predominantly internal epigenetic modification in eukaryotes mRNA, heralded the creation of the field of epi-transcriptomics. This post-transcriptional RNA modification is dynamic and reversible, and is regulated by methylases, demethylases and proteins that preferentially recognize m6A modifications. Altered m6A levels affect RNA processing, degradation and translation, thereby disrupting gene expression and key cellular processes, ultimately resulting in tumor initiation and progression. Furthermore, inhibitors and regulators of m6A-related factors have been explored as therapeutic approaches for treating cancer. In the present review, the mechanisms of m6A RNA modification, the clinicopathological relevance of m6A alterations, the type and frequency of alterations and the multiple functions it regulates in different types of cancer are discussed.

## The N6-methyladenosine (m6A) RNA modification

**Fig. 1:**
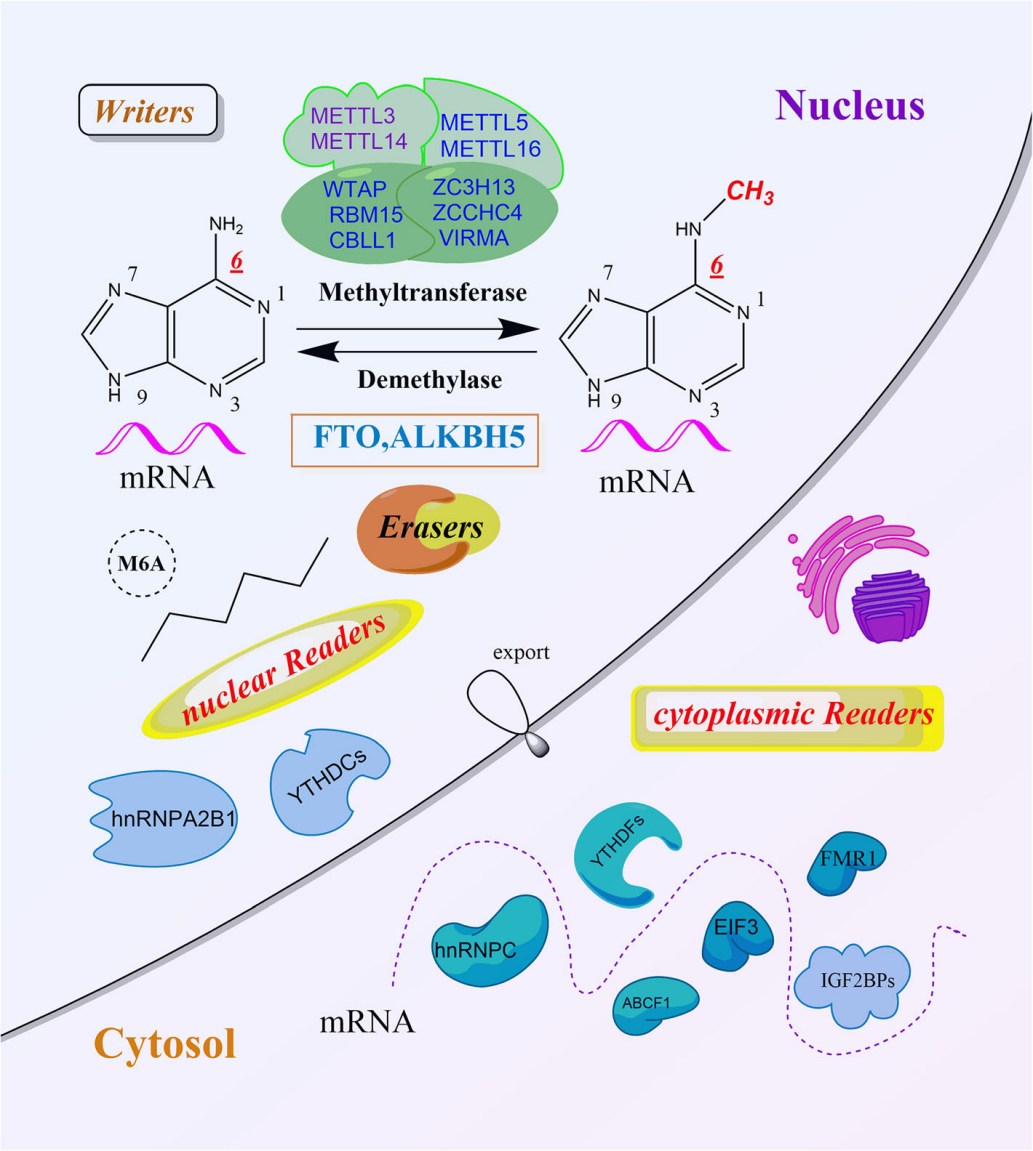
Chemical basis and molecular composition of m6A RNA methylation

M6A RNA modification, describes a methylation at the N6 position of adenosine, and is the most abundant internal modification in eukaryotes mRNA [1]. Since its discovery in 1974 [1], research on m6A has flourished owing to improvements in detection methods and the identification of important regulatory proteins and it recently reported that m6A modifications regulate the generation and function of transfer RNA (tRNA), ribosomal RNA (rRNA) and non-coding RNAs (ncRNAs), such as microRNA (miRNAs), long non-coding RNA (lncRNAs), and circular RNAs (circRNAs). Gene examination technology and high-throughput sequencing methods have demonstrated that the m6A modification is not randomly distributed, but is enriched near stop codons and 3′-untranslated terminal regions (UTRs) and translated near 5′-UTR or in long exons [2]. The m6A modification of RNA is dynamically and reversibly regulated by two important catalytic proteins, demethylases (writers) and methyltransferases (erasers) [3]. It is also recognized by a group of binding proteins so-called “readers” that decode m6A methylation and mediate recruitment of downstream functional complexes. A summary of known the machinery regulating m6A modifications is shown in Fig. 1.

M6A modifications influence RNA maturation, transcription, localization, translation and metabolism [4]. The biological significance of m6A is demonstrated by the vital molecular functions regulated by this modification in mammals, including nervous system development, the circadian rhythm, DNA damage response, heat shock response and tumorigenesis amongst others [5]. Furthermore, m6A regulators are tightly correlated with the activation and inhibition of cancer-associated signaling pathways. In the present review, a summary of the literature and hypotheses surrounding m6A modifications is provided with a focus on the functional mechanisms of this ubiquitous RNA modification in carcinogenesis. Additionally, the mechanisms underlying therapeutic approaches which target m6A regulators for the development of anti-cancer drugs are described.

### The methyltransferase complex writes the m6A modification

The m6A methyltransferase of RNA consists of “writer” proteins including: METTL3, METTL5, METTL14, METTL16 and their cofactors Wilms tumor 1associated protein (WTAP), RNA-binding motif protein 15 (RBM15/15B), Cbl proto-oncogene-like 1 (CBLL1; also known as HAKAI), zinc finger CCCH-type containing 13 (ZC3H13) and Vir-like m6A methyltransferase-associated (VIRMA; also known as KIAA1429). In 1997, METTL3 was demonstrated to serve as the primary methyltransferase critical for m6A methylation and aberrant expression of METTL3 could alter the total m6A methylation levels [6]. METTL14 serves as structural support for METTL3, and together they form the core methyltransferase complex inducing m6A modification synergistically [7]. WTAP stabilizes the core complex and promotes m6A by recruiting the complex to nuclear speckles [8]. RBM15/15B functions to assist binding of METTL3 and WTAP, directing the two proteins to their target sites [9]. VIRMA preferentially locates mRNA methylation modifications near the 3′-UTR and stop codon regions [10]. Other proteins, such as ZC3H13 and CBLL1, in concert with additional cofactors, including WTAP, control nuclear m6A methylation [11]. Recently, ZCCHC4, another CCHC zinc-finger-containing protein, was identified as a novel methyltransferase which was involved in the modification of the 28S rRNA, mediating rRNA ribosome subunit distribution and global translation [12].

METTL16 was proposed to act as an independent mRNA methyltransferase in 2017 [13]. It may regulate mRNA stability and splicing and its binding sites do not overlap with those of METTL3/METTL14 methylation complexes, suggesting that it functions independently [14]. Accordingly, METTL16 was confirmed to initiate splicing when a construct of METTL16 with a mutated catalytic domain was overexpressed [15]. Additionally, METTL16 could function alone and catalyze m6A on U6 snRNA and regulate tumorigenesis by targeting pre-mRNAs and ncRNAs [13, 16]. However, there are few studies which have shown that METTL3/16 may function as an m6A ‘reader’ [3]. Several writers, such as METTL3/16, are multifunctional enzymes with prominent non-catalytic activities. In the absence of an enzyme cofactor presence, m6A writers function as readers and bind to unmodified substrates constitutively, thus triggering non-catalytic functions [16]. Recently, METTL5 was identified as a novel methyltransferase responsible for 18S rRNA m6A modification [17, 18]. METTL5 forms a heterodimer with TRMT112 increasing its metabolic stability and modification area on precursor and mature forms of 18S rRNA. Similar to the complex of METTL3/METTL14, TRMT112 is a coactivator of METTL5. The atomic resolution structure of METTL5-TRMT112, supports the hypothesis that its RNA-binding mode differs distinctly from that of other m6A writers [17].

Additionally, 26 core interacting factors amongst hundreds of WTAP-binding proteins have been identified by co-immunoprecipitation studies, and > 100 proteins may bind to METTL3 or METTL14 [19]. Thus, there may be other components of the m6A methyltransferase complexes which remain to be discovered.

### M6A methylation is removed via specific demethylases

Unlike the large multi-subunit m6A methyltransferase complex, only two m6A demethylases, FTO and AlkB homolog (ALKBH)5, have been identified. The two proteins are predominantly localized in the nucleus where the removal of m6A modification occurs. As a member of the AlkB family with a well-conserved catalytic domain, FTO was the first protein to be identified to catalyze m6A demethylation [20] and the notion of reversible m6A methylation in RNA was described. The hypothesis that FTO affects human obesity resulted in interest in examining its function [21]. ALKBH5 was the second RNA demethylase to be identified that could oxidatively reverse m6A modifications. ALKBH5 is expressed in the majority of the tissues, and its expression is particularly abundant in the testes [22]. Recently, the unique crystal structure of ALKBH5 have been resolved by several groups [22, 23]. Remarkably, FTO could mediate m6Am (N6,20-O-dimethyladenosine) demethylation as well. Unlike FTO, ALKBH5 seems to be an m6A-specific demethylase in mRNA [24]. These findings have greatly facilitated the development of inhibitors of m6A demethylases.

In addition, recent studies have shown that ALKBH3 may serve as a novel demethylase of m6A modifications [25, 26]. They identified m6A in mammalian tRNA as a novel ALKBH3 substrate and ALKBH3 preferentially modifies tRNA over mRNA or rRNA [25, 26].

#### M6A readers recognize m6A modification and confer specific phenotypic outcomes

“Readers” are comprised of YTH domain-containing proteins (YTHDF1/2/3 and YTHDC1/2), heterogeneous nuclear ribonucleoproteins (including hnRNPC, hnRNPG and hnRNPA2B1) and insulin-like growth factor 2 mRNA-binding proteins (IGF2BPs YTHDF1/2/3 and YTHDC1/2). In the cytosol, YTHDF1 interacts with initiation factors to facilitate RNA translation initiation [21]. YTHDF2 selectively binds m6A-methylated mRNA and regulates RNA degradation. YTHDF3 facilitates translation by promoting protein synthesis in synergy with YTHDF1 and affects mRNA decay mediated by YTHDF2 [27]. All three YTHDF proteins function co-operatively in fundamental biological pathways [28]. Moreover, YTHDF1 and YTHDF2 recognize circRNAs m6A marks and modify circRNAs expression [29]. YTHDC1 could increase the export of circNSUN2 to the cytoplasm [30]. In contrast to the functions of YTHDF2, IGF2BPs enhance the stability and translation of their target mRNAs by recognizing m6A modifications under normal and stressed conditions [31]. HNRNPC selectively recognizes m6A-induced splicing in mRNA secondary structures, whereas HNRNPA2B1 recognizes pri-miRNA m6A marks and interacts with DGCR8, thus stimulating miRNA processing [32].

Furthermore, several novel readers of m6A have been identified. In the cytoplasm, mRNA translation is also stimulated by the direct readers, eukaryotic initiation factor 3, Fragile X mental retardation 1 (FMR1) and ATP binding cassette subfamily F member 1 [33]. Taken together, the intricate interactions between m6A modifications and RNA-binding proteins may regulate mRNA expression at multiple levels.

## m6A in cancer

Numerous studies have confirmed the effects of m6A modifications and its ability to fine-tune and coordinate gene expression [5, 25, 34–39]. The alterations of m6A levels may profoundly influence cancer hallmarks, including sustaining proliferative signaling, evading growth suppressors, resisting cell death, enabling replicative immortality, inducing angiogenesis, activating invasion and metastasis, reprogramming energy metabolism, genomic instability and mutation, evading immune destruction and tumor-promoting inflammation, suggesting that m6A may function as an oncogenic or suppressive role in malignant tumors [25, 35, 37, 38, 40]. Certain proteins require m6A modification to participate in the mechanisms underlying the development of cancer, but it is not clear whether they make an effect in m6A modification or not. The specific roles of m6A proteins in human cancers are summarized in Tables 1 and 2.

**Table 1:** Oncogenic roles of m6A proteins and biological mechanisms exerted in human tumors

Cancer	Regulator	Role in cancer	Mechanism	Functional classification	Refs
AML	METTL3	Oncogene	Promote translation of MYC, BCL2 and PTEN	Inhibit differentiation of HSPCs, increase cancer cells growth and inhibit apoptosis	[41]
AML	METTL14	Oncogene	Stabilize MYC and MYB	Inhibit differentiation and promote leukemia cells self-renewal	[42]
AML	WTAP	Oncogene	Target rapamycin (mTOR) expression and PMA	Promote AML cells proliferation and block the differentiation	[43]
AML	FTO	Oncogene	Destabilize ASB2 and RARA	Promote leukemic oncogene-mediated cells transformation and leukemogenesis	[44] [45]
			Stabilize MYC and CEBPA	Increase proliferation/survival of cancer cells	
AML	YTHDF2	Oncogene	Stabilize mRNAs like Tal1	Inhibit HSCs expansion	[46]
AML	IGF2BP1	Oncogene	Form LIN28B/let-7/IGF2BP1 signaling axis	Increase leukemia cells growth and metabolism	[47]
GBM	METTL3	Oncogene	Upregulate SOX2	Attenuate differentiation, enhance DNA repair and tumor growth	[48]
GBM	FTO	Oncogene	Upregulate oncogenes like ADAM19/EPHA3/KLF4	Promote GSCs growth and self-renewal	[49]
GBM	ALKBH5	Oncogene	Promote tumorigenesis by stabilizing FOXM1 mRNA	Sustain tumor cells proliferation program	[50]
LC	METTL3	Oncogene	Enhance translation of EGFR and TAZ	Promote growth, survival, and invasion of cancer cells	[51] [52] [53] [54] [55]
			Targeted by miR-33a	Promote the proliferation of NSCLC cells	
			Enhance translation of BRD4 via eIF3	Promotes tumorigenicity	
			Promote YAP translation by regulating miR-1914-3p	Induce invasion and metastasis of NSCLC	
			Regulate miR-143-3p/VASH1 axis	Induce brain metastasis and angiogenesis	
LC	FTO	Oncogene	Enhance MZF1 expression and stabilize MZF1 transcript	Facilitate cancer cells proliferation and invasion	[56] [57]
			Strengthen the stability of USP7 mRNA	Promote cancer cells growth	
LC	YTHDF2	Oncogene	Facilitate METTL3-mediated SOCS2 m6A modification	Induce LC cells growth and metastasis	[58]
LC	IGF2BP1	Oncogene	Increase SRF stability	Promote tumor cells growth and enhance cells invasion	[59]
NPC	METTL3	Oncogene	Stress the ZNF750-FGF14 signaling axis	Promote NPC growth and inhibit cells apoptosis	[60]
HCC	METTL3	Oncogene	Promote SOCS2 degradation	Induce HCC cells proliferation, migration, and colony formation	[58] [61]
			Regulate EMT key translator Snail	Activate the migration, invasion and EMT of cancer cells	
HCC	KIAA1429	Oncogene	Inhibit ID2 mRNA	Facilitate the migration and invasion of cancer cells	[62] [63]
			Induce HuR separation and degrade GATA3 pre-mRNA	Induce the tumor growth and metastasis	
HCC	WTAP	Oncogene	Silence of ETS1 via m6A-HuR-dependent mechanism	Promote the proliferation capability and tumor growth of HCC cells	[64]
HCC	YTHDF2	Oncogene	Downregulate miR-145 Facilitate METTL3-mediated SOCS2 m6A modification	Promote proliferation of HCC cells Induce HCC cells proliferation, migration, and colony formation	[65] [58]
HCC	IGF2BP1	Oncogene	Increase SRF mRNA stability	Promote tumor cells growth and enhance cells invasion	[59]
HB	METTL3	Oncogene	Stabilize CTNNB1 via Wnt/β-catenin pathway	Promote the proliferation of HB	[66]
CRC	METTL3	Oncogene	Upregulate lncRNA RP11	Facilitate the migration, invasion and EMT of CRC cells	[67] [68] [69] [70]
			Maintain SOX2 expression via IGF2BP2	Sustain CRC cells self-renewal, stem cell frequency and migration	
			Regulate miR-1246/SPRED2/ MAPK signaling	Promote the metastasis and migration of CRC cells	
			Upregulate CBX8 assisted by IGF2BP1	Maintain the stemness properties of cancer cells	
CRC	FTO	Oncogene	Downregulate miR-1266	Promote the proliferation of CRC cells	[71] [72]
			Initiate cellular signaling molecules like STAT3	NM	
CRC	WTAP	Oncogene	Form WTAP-WT1-TBL1 axis	Inhibit cell apoptosis and cell cycle arrest and promote cell proliferation	[73]
CRC	YTHDC2	Oncogene	Upregulate HIF-1α	Activate cell metastasis	[74]
CRC	YTHDF1	Oncogene	Promoted by c-Myc	Promote the proliferation of CRC cells	[75] [76]
			Inhibit Wnt/β-catenin pathway activity	Promote the cell cycle progression and the tumorigenicity of CRC cells	
CRC	IGF2BP1	Oncogene	Bind CBX8 mRNA and promote CBX8 expression	Maintain the stemness properties of cancer cells	[70] [77] [31]
	IGF2BP2	Oncogene	Stabilized by lncRNA LINRIS	Promote tumor growth and the aerobic glycolysis in CRC	
	IGF2BPs	Oncogene	Promote MYC	Promote cell proliferation, colony formation ability, migration and invasion	
PDAC	METTL3	Oncogene	Promote miR-25-3p maturation and activation of AKT-p70S6K	Promote cell proliferation, migration, and invasion	[78]
PDAC	YTHDF2	Oncogene	Activate AKT pathway	Promote cell proliferation	[79]
GC	METTL3	Oncogene	Enhance HDGF mRNA	Promote proliferation, liver metastasis, tumor angiogenesis and glycolysis in GC	[80]
GC	ALKBH5	Oncogene	Decrease methylation of lncRNA NEAT1	Promote invasion and metastasis of GC	[81]
BCA	METTL3	Oncogene	Accelerate pri-miR221/222 maturation	Sustain tumor proliferation of BCA	[82] [83] [84]
			Form AFF4/NF-κB/MYC signaling axis	Promote BCA cell proliferation, invasion, tumorigenicity and survival	
			Promote CDCP1 translation	Promote malignant transformation of uroepithelial cells and BCA tumorigenesis	
PCA	YTHDF2	Oncogene	Target miR-493-3p	Promote PCA cells proliferation and migration	[85]
PCA	METTL3	Oncogene	Regulate hedgehog pathway	Facilitate cell proliferation, survival, colony formation, and invasion	[86]
RCC	WTAP	Oncogene	Enhance CDK2 expression	Enhance cell proliferation abilities	[87]
CSCC	FTO	Oncogene	Target β-catenin	Promote chemo-radiotherapy resistance of CSCC in vitro and in vivo	[88] [89]
			Promote transcripts of E2F1 and MYC	Facilitate cell proliferation and migration	
BC	METTL3	Oncogene	Form a positive feedback loop of METTL3/HBXIP/let-7 g	Promote cell proliferation and inhibit cell apoptosis	[90]
			Target BCL-2	Accelerate the proliferation, inhibit the apoptosis and the tumor growth	[91]
BC	ALKBH5	Oncogene	Stabilize NANOG and KLF4	Increase the percentage of BCSCs and phenocopy the effect of hypoxia	[92]
		Oncogene	Target TGFβ1 signaling–associated transcripts	Promote cell growth, invasion, inappropriate cell cycle activity and evasion of apoptosis	[93]
BC	FTO	Oncogene	Target BNIP3	Promote BC cells proliferation, colony formation and metastasis	[94]
EOC	METTL3	Oncogene	Upregulate AXL translation	Increase cellular proliferation, motility, invasion, and tumor formation and promote EMT	[95]
EOC	ALKBH5	Oncogene	Target miR-7 and BCL-2	Promote the proliferation and invasion in vitro and in vivo via inhibiting the autophagy	[96]
EOC	IGF2BP1	Oncogene	Sustain the expression of SRF-target oncogenes	Promote tumor cells growth and enhance cell invasion	[59]
Melanoma	FTO	Oncogene	Target PD-1, CXCR4, SOX10, CTSV2, and NOP16	Increase tumor growth and decrease response to anti-PD-1 blockade immunotherapy	[97]
cSCC	METTL3	Oncogene	Promote △Np63 expression	Promote cSCC cell stem-like properties like colony forming ability and tumorigenicity	[98]
EBV	METTL14	Oncogene	EBNA3C hijacks METTL14	Induce proliferation and colony formation of EBV positive cells	[99]

*AML* acute myeloid leukemia; *GBM* glioblastoma; *LC* lung cancer; *NPC* nasopharyngeal carcinoma; *HCC* hepatocellular carcinoma; *HB* hepatoblastoma; *CRC* colorectal cancer; *PDAC* pancreatic cancer; *GC* gastric carcinoma; *BCA* bladder cancer; *PCA* prostate cancer; *RCC* renal cell carcinoma; *CSCC* cervical squamous cell carcinom; *BC* breast cancer; *EOC* epithelial ovarian cancer; *cSCC* cutaneous squamous cell carcinoma; *EBV* EBV-associated cancer; *NM* not mentioned

**Table 2:** Suppressive roles of m6A proteins and biological mechanisms exerted in human tumors

Cancer	Regulator	Role in cancer	Mechanism	Functional classification	Refs
GBM	METTL3 METTL14	Suppressor	Downregulate oncogenes like ADAM19	Suppress GSCs growth and self-renewal	[49]
HCC	METTL14	Suppressor	Interact with DGCR8 and modulate the primary miR-126 process	Suppress tumor invasion and metastasis	[100]
HCC	YTHDF2	Suppressor	Inhibit STAT3 phosphorylation by degrading IL11 and SERPINE2 mRNA; Activate MEK/ERK pathway, destabilizing EGFR mRNA	Inhibit inflammation, vascular reconstruction and metastatic progression	[101] [102]
CRC	METTL3	Suppressor	Regulate p38/ERK pathways	Suppress CRC cancer proliferation and migration	[103]
CRC	METTL14	Suppressor	Regulate primary miR-375 processing	Inhibit CRC cell growth and metastasis	[104]
PDAC	ALKBH5	Suppressor	Demethylate lncRNA KCNK15-AS1	Inhibit pancreatic cancer motility and EMT	[105]
PDAC	YTHDF2	Suppressor	Destabilize YAP mRNA	Inhibit cancer migration, invasion, and adhesion ability	[79]
BCA	METTL14	Suppressor	Target Notch1	Inhibit bladder TIC self-renewal and bladder tumorigenesis	[106]
RCC	METTL3	Suppressor	Change EMT and PI3K-Akt-mTOR pathways	Suppress proliferation, migration, invasion function and cell cycle of RCC	[107]
RCC	FTO	Suppressor	Increase expression of PGC-1α	Impair tumor growth and induce apoptosis via regulating mitochondrial biogenesis and oxidative phosphorylation	[108]
EC	METTL3/METTL14 METTL14	Suppressor	Active AKT signaling pathway	Inhibit the proliferation and tumorigenicity of in vitro and in vivo	[109]
Melanoma	YTHDF1	Suppressor	Bind HINT2	Restrain cell growth and migratory ability	[110]

*GBM* glioblastoma; *HCC* hepatocellular carcinoma; *CRC* colorectal cancer; *PDAC* pancreatic cancer; *BCA* bladder cancer; *RCC* renal cell carcinoma; *EC* Endometrial cancer

### Hematological malignancy: acute myeloid leukemia (AML)

AML is the result of uncontrolled proliferation and defects in cell differentiation of myeloid white blood cells, with distinct genetic aberrations, for which the therapeutic options remain unsatisfactory [111]. Mechanistically, several studies have shown that MELLT3 and METTL14 serve an oncogenic role in AML by promoting the translation of MYC, MYB, BCL2, SP1 and PTEN, thus increasing the levels of phospho-AKT [41, 42]. Additionally, METTL3 has been also shown to be mis-localized in the cytoplasm and results in a concomitant increase in WTAP expression, and WTAP has been demonstrated to function as a tumor suppressor gene. However, Bansal et al identified the oncogenic role of WTAP and its target, which is involved in the mTOR signaling pathway, in AML [43]. RBM15 exhibits a well-established oncogenic role in the development of hematologic malignancies [112] and is a fusion partner of the MKL1 gene in acute megakaryoblastic leukemia, a subtype of pediatric AML [113]. Notably, FTO expression is increased in AML with t(11q23)/MLL rearrangements, t(15; 17)/PML-RARA, FLT3-ITD, and/or NPM1 mutations. Downregulation of FTO inhibits the proliferation and differentiation capacity through reducing the abundance of m6A on the transcripts of ASB2 and RARA [44]. As core readers, YTHDFs and IGF2BPs may mediate the majority of the resultant phenotypes through regulation of MYC [46]. IGF2BP1 is a novel downstream target of LIN28B and functions via miRNA let-7 in AML, thus leading to cell cycle arrest, inhibition of cell proliferation and colony formation [47].

These studies corroborate the significance of m6A in AML. The regulators of m6A are all oncogenic in AML. METTL3, METTL14 and RBM15 expression are all upregulated in AML compared with other types of cancer [112]. Intriguingly, writers and erasers both serve a synergistic role in AML, and this may be due to the FTO-targeted sites, which exhibit effects on mRNA distinct from the known reading processes [114]. In previous studies, expression of METTL3, METTL14, FTO and YTHDFs were all correlated with MYC, highlighting the importance of precise regulation of MYC, and the notable impact dysregulation of MYC has on tumorigenesis [41, 42, 44, 46, 115]. Furthermore, hematopoietic stem cells (HSCs) notably influence AML. Abnormal or blocked differentiation of HSCs is a shared feature in AML. M6A regulates symmetric division of HSCs by modulating MYC mRNA levels, which is required for rapid regeneration during tissue damage and stress [116]. Mouse HSCs with METTL14 deleted obtained from primary leukemia blasts exhibit significantly delayed AML onset when implanted in mice [42]. RBM15 directly binds to and controls the differentiation of HSCs by regulating genes, such as GATA1, RUNX1, c-MPL and TAL1, which are critical for HSC self-renewal [113]. Suppression of YTHDF2 promotes expansion of HSCs ex vivo by stabilizing Tal1 mRNAs [46]. Therefore, focusing on HSCs or inhibition of MYC may serve as potential targets for treatment of AML.

### Neurological tumors: glioblastoma (GBM)

GBM is the most lethal type of primary brain tumor. Studies on the role of METTL3 in GBM have produced contradictory results. Initially, Cui et al demonstrated that METTL3 and METTL14 inhibited growth and tumorigenesis of glioblastoma stem-like cells (GSCs) by downregulating the ADAM19/EPHA3/KLF4 pathway [49]. However, the same year, another group produced contradictory results which showed that METTL3 promoted GSC growth by upregulating SOX2 expression and protected GSCs from radiation-induced cytotoxicity [48]. Other studies have proposed that FTO and ALKBH5 expression are associated with a less favorable prognosis in patients with GBM. The lncRNA antisense to FOXM1 promotes an interaction between ALKBH5 and FOXM1 and subsequently ALKBH5 demethylates FOXM1 nascent transcripts, enhancing FOXM1 expression, thus maintaining tumorigenicity of GBM [50]. FTO constrains the progression of GBM progression and significantly shortens the lifespan of GSC-grafted mice [49].

The phenotypic differences associated with METTL3 may be explained by a differing reliance on m6A-modified RNAs in different types of GBM cells and differences in genetic heterogeneity. In addition, the mechanisms by which METTL3 exerts its effects can be divided into two modes: m6A-dependent and m6A-independent. METTL3 may exert oncogenic functions independent of its catalytic activity or its downstream readers. Consequently, METTL3 itself or possibly an unknown METTL3-complex components may function as m6A reader proteins, and this may underlie the dual functions of METTL3 under specific conditions [51]. Notably, GSCs are almost ubiquitously used in studies regarding GBM. GSCs can self-renew, are resistant to conventional therapy and in in vivo models, they give rise to tumor recurrence [117]. These findings may pave avenues for developing effective therapeutic strategies for treatment of GBM [50].

### Respiratory tumors: lung cancer and nasopharyngeal carcinoma (NPC)

Lung cancer is the major cause of cancer-associated mortality worldwide. METTL3 acts as an oncogene in lung cancer via different mechanisms. METTL3 enhances translation of epidermal growth factor receptor (EGFR), the Hippo pathway effector TAZ and MAPKAPK2 (MK2) [51]. MiR-33a suppresses proliferation of non-small cell lung cancer cells via reducing the expression of METTL3 [52]. Additionally, METTL3 promotes YAP translation, increasing YAP activity via miR-1914-3p to induce drug resistance and metastasis [54]. Also, METTL3 facilitates the biogenesis of miR-143-3p to promote the brain metastasis of lung cancer via regulation of VASH1 [55]. In lung squamous cell carcinoma, METTL3 interacts with eukaryotic translation initiation 3 h to accelerate tumorigenicity by promoting translation of oncogenic mRNAs, such as Bromodomain-containing protein 4 (BRD4) [53]. Sumoylation of METTL3 also enhances tumorigenesis [118]. FTO expression is associated with a less favorable poor prognosis by increasing the expression levels of myeloid zinc finger protein 1 expression and the stability of ubiquitin-specific protease mRNA [56, 57]. Among the m6A readers, YTHDF2 facilitates METTL3-induced oncogenic effects by increasing degradation of SOCS2 [58]. IGF2BP1 is associated with a less favorable prognosis by increasing serum response factor mRNA stability and promoting cancer phenotypes in lung cancer [59].

In NPC, METTL3 is negatively associated with tumor repressor ZNF750, which is part of a ZNF750-FGF14 signaling axis that inhibits NPC growth [60]. LncRNA FAM225A, where m6A levels are highly enriched, functions as a competing endogenous RNA (ceRNA) sponging miR-590-3p and miR-1275, leading to the activation of FAK/PI3K/AKT signaling to promote proliferation and invasion of NPC cells [119]. Taken together, METTL3 governs in respiratory tumors. Moreover, m6A proteins could affect the biogenesis process of miRNAs/lncRNAs eventually influencing on development of tumor.

### Gastrointestinal tumors: hepatocellular carcinoma (HCC), colorectal cancer (CRC), pancreas cancer and gastric carcinoma (GC)

HCC is a significant public burden, and the incidence is rising worldwide [120]. As mentioned above, METTL3 and METTL14 exert an oncogenic role in HCC via YTHDF2-dependent post-transcriptional silencing of SOCS2 [51]. METTL3 and YTHDF1 act as opposing prognostic factors of overall survival of patients with HCC via regulation of Snail, a key translator of EMT [61]. Additionally, KIAA1429 facilitates migration and invasion of HCC by inhibiting ID2 [62]. GATA3-AS functions as a guide lncRNA that promotes a malignant phenotype driven by KIAA1429 [63]. WTAP promotes the proliferative capacity of HCC through a p21/p27-dependent pattern mediated by ETS proto-oncogene 1(ETSI) [64]. However, Ma et al demonstrated that METTL14 is an anti-metastatic factor, positively modulating DGCR8 binding to primary miR126 (pri-miR126) [100]. Amongst the m6A readers, YTHDF1 overexpression is associated with a poor prognosis in HCC [121] and YTHDF2 is closely associated with the malignancy of HCC through interactions with miR-145 [65]. In contrast, two groups have shown that YTHDF2 suppresses the development of HCC development through stabilization of EGFR or interleukin 11 mRNA [102]. YTHDF2 downregulation increased inflammation and abnormal vascularization, degrading the mRNA of tumor suppressor genes in HCC [101]. In cholangiocarcinoma, WTAP is correlated with HCC metastasis [122]. In hepatoblastoma, METTL3 promotes development of hepatoblastoma development through increasing the expression of CTNNB1 via regulation of the Wnt/β-catenin pathway [66].

CRC has the second highest incidence of death worldwide [123]. METTL3 exhibits dual roles in CRC. METTL3 increases the expression of lncRNA RP11, which subsequently stimulates Zeb1 expression, initiating the dissemination of CRC cells [67]. Li et al demonstrated that METTL3 facilitates tumor progression via maintenance of expression of the stem cell marker SOX2, in an IGF2BP2-dependent manner in CRC [68]. They also suggested that METTL3 may serve as a marker of cancer stem cells (CSCs) due to its role in promoting stemness. Meanwhile, METTL3-mediated m6A modification and IGF2BP1 binding directly to CBX8 mRNA both could induce aberrant overexpression of CBX8, thus maintaining the stemness and inhibiting the chemosensitivity of CRC [70]. Peng et al confirmed that METTL3 advances the maturation of pri-miR-1246, where it further reverses the inhibition of the MAPK pathway, thus promoting metastasis [69]. However, recently METTL3 and METTL14 were reported to proliferation and migration of suppress CRC through regulating the p38/ERK pathway and tumor suppressor miR-375, respectively [103, 104]. Zhang et al showed that WTAP was a novel oncogene in CRC by Wnt signaling pathway [73]. In regards to erasers, FTO promoted progression of CRC cells through degrading expression of miR-1266, or initiation of the cellular signaling molecules STAT3, cyclin D1 and MMPs [71, 72]. YTHDC2, YTHDF1 and IGF2BPs are all hypothesized to promote metastasis of CRC by upregulating HIF-1α or c-Myc expression [31, 74, 75]. Yang *et a* showed that the specific mechanism by which YTHDF1 functions in CRC was through inhibition of the Wnt/β-catenin pathway, thus accelerating tumorigenicity and CSC activity [76]. Most recently, Wang et al introduced that lncRNA LINRIS stabilizes IGF2BP2 and promotes progression of CRC via aerobic glycolysis pathway [77].

Pancreatic cancer is a lethal malignancy, and is one of the most aggressive types of cancer [124]. Chen et al showed that YTHDF2 performed dual cellular functions in pancreatic cancer cells [79]: Promoting proliferation and inhibiting migration via different pathways, forming a phenomenon termed the migration-proliferation dichotomy. A novel mechanism was unveiled by which ALKBH5 inhibits the motility of pancreatic cancer by demethylating lncRNA KCNK15-AS1 [105]. Cigarette smoke condensate promotes aberrant overexpression of METTL3 in smokers, significantly promoting maturation of the oncogene, primary miR-25-3p, which activates AKT-p70S6K oncogenic signaling [78]. Bioinformatics analysis drew a consistent conclusion that METTL3 and FTO may promote proliferation and invasion of pancreatic cancer [125].

Despite the decline in the death rate of patients with GC, it is still the fifth most common malignancy worldwide [126]. miR-660 reduces proliferation by regulating expression of the oncogene E2F3 via m6A modifications in GC [127]. METTL3 promotes GC angiogenesis and glycolysis by increasing the stability of HDGF mRNA and activating the AKT signaling pathway, respectively [80]. ALKBH5 promotes invasion and metastasis of GC by decreasing methylation of the lncRNA NEAT1 [81]. Bioinformatics analysis predicted that m6A suppression promotes GC development through activating the Wnt/PI3K-AKT signaling pathway, whereas increasing m6A levels reversed these phenotypical and molecular changes [128, 129].

Cumulatively, emerging studies have focused on gastrointestinal tumors in 2019. These findings highlight the interaction between miRNAs/lncRNAs with m6A proteins in gastrointestinal tumors, such as pri-miR-126, miR-145, miR-1266, miR-1246, miR-25-3p, lncRNA NEAT1, KCNK15-AS1 and GATA3-AS. m6A promotes tumorigenesis via dysregulation of miRNAs/lncRNAs to modulate metastatic progression and increasing chromosomal instability [130, 131]. For example, METTL3 promotes the maturation of miRNAs such as let-7e, miR221/222, miR-4485, miR-25, miR-93, miR-126, miR-1246 and miR-335. METTL16 is associated with various ncRNAs, lncRNAs and pre-mRNAs, including MALAT1 lncRNA. IGF2BP1 enhances an aggressive phenotype in tumor cells by impairing miRNA-directed downregulation of oncogenic factors [132]. Therefore, further identification of tumor-related miRNAs/lncRNAs and investigations of their functions may highlight other interactions where m6A modifications are involved. CSCs and oncogene MYC exert powerful effects on gastrointestinal tumors, similar to those observed in GBM. METTL14 and YTHDF2 however, exert the opposite effect to that observed in HCC, and the same is true of the METTL3 and CRC. These targets may highlight potentially effective therapeutic strategies for treatment of gastrointestinal tumors.

### Urological tumors: bladder cancer (BCA), renal cell carcinoma (RCC) and prostate cancer (PCA)

In 2019, several groups explored the function of m6A in bladder cancer [82–84, 106]. Cheng et al showed that METTL3 promoted the progression of BCA via an AFF4/NF-κB/MYC signaling network [83]. Shortly after, other groups showed that METTL3 promoted proliferation of BCA cells by accelerating pri-miR221/222 maturation and upregulating the expression of the oncogene CDCP1 [82, 84]. Bioinformatics analysis showed that m6A RNA methylation regulators can contribute to the malignant progression of BCA [133]. Gu et al demonstrated that METTL14 inhibited the self-renewal capacity of BCA initiating cells through targeting Notch1 [106]. These recent studies provide novel insights into new avenues for BCA therapy, and determining the inter-associations between the different underlying mechanisms may facilitate this.

Relatively fewer studies have been reported on the role of m6A modifications in PCA. METTL3 silencing decreases expression of GLI1, an important apoptotic factor involved in the hedgehog pathway [86]. YTHDF2 and miR-493-3p are cited as two crucial oncogenes, involved in the progression of PCA by indirectly modulating m6A levels [85].

Among urological malignancies, RCC is the most lethal [126]. Methylenetetrahydrofolate dehydrogenase 2 overexpression enhances m6A modification of HIF-2α and forms a positive feedforward loop in RCC, resulting in malignant phenotypes [134]. Li et al demonstrated that METTL3 could suppress proliferation, migration and epithelial-to-mesenchymal transition (EMT) of RCC cells via regulation of the PI3K-AKT-mTOR pathway [107]. METTL14 inhibits P2RX6 protein translation and modulates ATP-P2RX6-Ca^2+^-p-ERK1/2-MMP9 signaling to prevent migration and invasion of RCC cells [135]. Additionally, WTAP promotes tumorigenesis by enhancing CDK2 expression [87] and FTO is expression is decreased in clear cell RCC, reducing tumor growth via increasing the expression of PGC-1α, a central regulator of mitochondrial function in the PPARγ co-activator family [108].

### Gynecological oncology: breast cancer (BC), cervical squamous cell carcinoma (CSCC), epithelial ovarian cancer (EOC) and endometrial cancer (EC)

Breast cancer is the most common type of cancer in women worldwide [136]. Cai et al showed that METTL3 increases the expression of mammalian hepatitis B X-interacting protein (HBXIP), thus driving the aggressiveness of BC. HBXIP upregulates the expression of METTL3 via inhibiting the function of the tumor suppressor miRNA let-7 g, forming a positive feedback loop of METTL3/HBXIP/let-7 g/METTL3 [90]. Consistent with this, a recent study showed that METTL3 promoted BC progression by targeting BCL-2 [91]. Hypoxia induces ALKBH5 to demethylate NANOG mRNA and enhance its stability in BC stem cells (BCSCs) [92]. The group further demonstrated that ZNF217 and ALKBH5 play complementary roles in negatively regulating m6A levels, eventually increasing the number of BCSCs under hypoxic conditions [137]. Furthermore, ALKBH5 and METTL14 interact with each other and inhibit YTHDF3 activity, thus accelerating tumor angiogenesis. They stated that METTL14 and ALKBH5 constitute a positive feedback loop with HuR to regulate the target genes of cell cycle progression, EMT and angiogenesis [93]. In 2019, Jessica et al proposed that through regulating m6A methylation, far upstream binding protein 1 (FUBP1) globally affects alternative splicing to promote the activity of proteins associated with BC neoplastic transformation, including BRCA1, MAGI3 and CASP8 [138]. Niu et al showed that FTO promoted tumor development via inhibiting BNIP3, a pro-apoptotic gene of the BCL-2 family [94]. Notably, members of the BCL-2 family have repeatedly been shown to involved in the development and progression of BC, and are targeted by FTO and METTL3 [91]. The translation process of BCL-2 is also promoted by METTL3 in AML and ALKBH5 in epithelial ovarian cancer [41, 96]. Thus, m6A proteins may act to inhibit the activity of members of the BCL-2 family at various stages of the BCL-2 signaling process, thereby providing a favorable therapeutic response.

M6A also serves a role in several other types of gynecological cancer. In CSCC, FTO enhances chemoradiotherapy resistance by targeting β-catenin [88]. FTO also interacts with the transcripts of E2F1 and MYC to facilitate proliferation and migration [89]. In EC, Liu et al demonstrated that reduced levels of METTL3/METTL14 and an accumulation of FTO induced by estrogen enhanced AKT/mTOR signaling to promote tumorigenicity [109]. In EOC, METTL3 promotes tumorigenicity through regulating translation of AXL and EMT [95]. ALKBH5 acts as a candidate oncogene, inhibiting cancer autophagy through miR-7 and BCL-2, eventually activating an EGFR-PI3K-AKT-mTOR signaling pathway [96]. The PI3K/AKT/mTOR pathway has been implicated in the development of various types of cancer regulated by m6A proteins, including METTL3/WTAP in AML [41, 43], METTL3 in RCC/PDAC [78, 107], ALKBH5 in EOC [96], and FTO in melanoma [97] and EC [109]. YTHDF2 and RBM15 expression are also correlated with activation of this pathway [139]. Activation of this pathway in AML and EC may be inhibited by rapamycin, an mTOR specific inhibitor. These data suggest that preventing communication between mTOR signaling and m6A regulators may present a potential avenue for treatment of various types of cancer.

### Skin neoplasm: melanoma and cutaneous squamous cell carcinoma (cSCC)

Melanoma is notorious for its high rate of mortality and its resistance to available therapies [140]. In 2019, two groups probed for the mechanisms underlying development and progression of melanoma [97, 110]. YTHDF1 suppresses ocular melanoma through modulation of mRNA translation of histidine triad nucleotide-binding protein 2, a tumor suppressor in ocular melanoma [110]. Another group showed that induction of FTO promotes tumorigenicity via mTOR signaling through m6A-mediated tuning of the PD-1 gene. Subsequently, IFN-γ downregulates FTO expression and may mediate the effect of FTO knockdown in PD-1 blockade [97]. In this study, m6A effective proteins influence the immune response by controlling signal transduction. Immune checkpoint blockade therapy has demonstrated an unprecedented anti-tumor response rate in patients with advanced cancer. Therefore, the complete mechanism of immune regulation by PD-1 blockade with m6A modifications in melanoma should be determined. Additionally, in cSCC, METTL3 upregulates ΔNp63 expression to promote tumorigenesis [98].

## The landscape of alterations of m6A regulators in human cancers

**Fig. 2:**
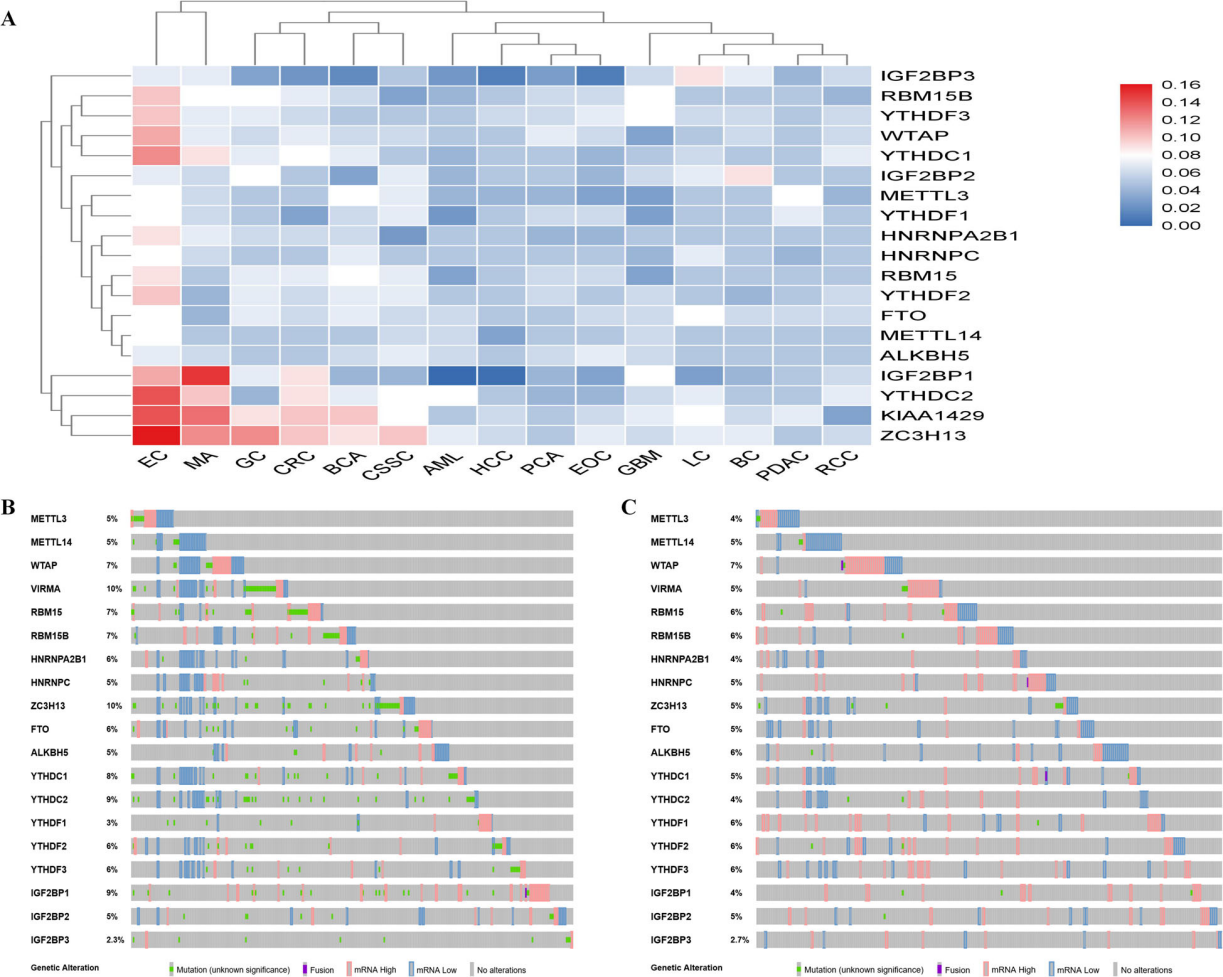
The alterations frequency of m6A regulators in cancers from cBioPortal data analysis. Totally 15 different TCGA projects were included (TCGA, PanCancer Atlas), and each project represents a specific cancer type. Oncoprints in cBioPortal were also used to represent the proportion and distribution of samples with altered m6A regulators. **a** The overall alterations frequency of m6A regulators across 15 cancer types. **b** The type and percentages of m6A regulators alterations in CRC. **c**The type and percentages of m6A regulators alterations in PCA

The vast majority of existing studies have focused on the m6A perturbation mediated via knockdown or overexpression of m6A related protein in cell death, proliferation, impaired self-renewal capacity and developmental defects. Meanwhile, mutations may cause gain or loss of m6A sites, thus affecting cellular m6A modification and associated with human cancers [24]. So, we investigated the alterations frequency (overexpression, down-regulation and mutation) of m6A proteins in cancers based on the cBioPortal database. The overall average alterations frequency of m6A proteins ranged from 0 to 16%. M6A related proteins exhibited a relatively higher alterations frequency in EC and melanoma. Besides, among m6A proteins, ZC3H13, KIAA1429, YTHDC2and IGF2BP1 showed higher alterations frequencies while m6A erasers seldom had a genetic mutation (Fig. 2a). Moreover, m6A proteins exhibited few alterations in several cancer types like PCA and AML (Fig. 2a). All data were collected from the cBioPortal database (Additional file 1: Table S1). The overexpression of m6A proteins was more frequent than the down-expression and mutation, which indicated that m6A proteins always served as an oncogenic role in cancers. To exemplify, METT14 alteration made up about 5% in CRC and the down-expression of METT14 mainly occurred in CRC (Fig. 2b), which is consistent with the previous reports [141]. Besides, in PCA, the incidence of IGF2BP1 alteration was 4% and it is the overexpression of IGF2BP1 rather than the down-expression or mutation that mainly occurred (Fig. 2c). The type of alterations m6A proteins in other cancers were also provided (Additional file 2: Figure S1).

Li et al systematically studied the mutation of m6A regulators across 33 cancer types and they found that the average mutation frequency of m6A regulators was low, ranging from 0.02–8.07%. M6A regulators exhibited a relatively higher mutation frequency of EC and melanoma. YTHDC1, IGF2BP1, YTHDC2, FTO and the writers showed relatively higher mutation frequencies [139]. While in AML, mutations of m6A regulatory genes were low (2.6%) and were significantly associated with poorer cytogenetic and genotypes risk, predicting poorer OS and EFS independently [142]. As well, Wu et al found genetic mutations of m6A enzymes occurred 24% of 2051 patients with BC. Nevertheless, the reduced level of the m6A members METTL3, METTL14, WTAP and FTO but not their mutation and overexpression was tightly associated with poor survival [143]. Zhang et al illustrated that mutations of METTL3, METTL14, ALKBH5, FTO, YTHDF1, YTHDF2 and YTHDF3 were rare in GC. The content and functions of m6A in GC might be impaired by specific mutations, thus predicting malignant phenotypes and augmenting Wnt/PI3K-Akt signaling in GC [144]. Liu et al found that the hotspot R298P mutation in METTL14 was more prevalent than other mutations in EC and occurred in ~ 1.5% of EC patients. The mutation eventually regulated AKT activity to promote the proliferation and tumorigenicity of EC [109]. In GBM, the genetic change (mutation or copy number variations) frequencies of the m6A RNA methylation regulators were very low (all≤1.1%) and the expression changes of these regulators were not caused by the genetic changes of the corresponding genes [145]. The mutations of m6A genes in several cancers were summarized in Table 3.

**Table 3:** The mutations of m6A genes in several cancers

Regulators	Mutation frequency	Cancer	Role	Data source	Effect/ Mechanism on cancer	Refs
METTL3/METTL14/YTHDF1/YTHDF2/FTO/ALKBH5	2.60%	AML	Oncogene	cBioPortal	Predict poorer OS and EFS independently	[142]
all included m6A enzymes	24%	BC	NM	cBioPortal/METABRIC	NM	[143]
all included m6A enzymes	23.33%	GC	Oncogene	TCGA	Predict malignant phenotypes and augmenting Wnt/PI3K-Akt signaling	[144]
METTL14	1.50%	EC	Oncogene	cBioPortal	Regulate AKT activity to promote tumorigenicity	[109]

*AML* acute myeloid leukemia; *BC* breast-invasive carcinoma; *GC* gastric carcinoma; *EC* Endometrial cancer; *NM* not mentioned

These results revealed a highly heterogeneous genetic and expression alteration landscape of m6A regulators across cancers. The landscape of alterations m6A regulators regulating in tumors laid a critical foundation for understanding the dysregulation of RNA methylation.

## The clinicopathological relevance to m6A alterations in tumors

The clinicopathological features resulted from m6A alterations are quite vital and could further provide us the development of drugs against m6A related proteins for cancer treatment. Most included studies illustrated that m6A related proteins may influence the prognosis of cancer patients. Overall, the high level of m6A methylation would lead to poor prognosis. However, only a few reports are available where other clinicopathological features like metastasis and invasion were provided. For example, in lung cancer, patients with high expression of METTL3 were prone to occur lymph node metastasis and distant metastasis [54]. Ma et al indicated that aberrant expression of METTL14 mRNA was correlated not only with tumor differentiation and tumor stage but also with tumor encapsulation and microvascular invasion, possibly playing a suppressive role in HCC metastasis [100]. Reduced m6A modification demonstrated adverse clinical outcomes in GC [144]. In bladder cancer, patients with high expression of METTL3 had worse prognosis and shorter survival time, compared with those with low expression of METTL3 [82]. The FTO expression was markedly declined in cancer counterpart and lost in the later stage. The reduced expression of FTO was correlated with worse OS and DFS [108]. These observed clinical changes might provide alternative, promising therapeutic targets for the treatment of m6A relevant cancers. The clinicopathological information regarding m6A proteins alterations was summarized in Table 4.

**Table 4:** The m6A protein alterations are correlated with clinicopathological features

Cancer	Regulator	Alteration	Prognosis	Metastasis/Invasion	Tumor size	Tumor stage	Histological grade	Recurrence	Therapy	Refs
AML	METTL3/ WTAP	Overexpression	Poor							[41, 43]
AML	METTL3/METTL14/YTHDF1/YTHDF2/FTO/ALKBH5	Mutation	Poor							[142]
GBM	METTL3	Overexpression	Poor						Radioresistance	[48]
GBM	WTAP/RBM15/YTHDF1/ ALBKH5	Overexpression	Poor				Increase			[49]
LC	METTL3	Overexpression	Poor	Lymph node metastasis Brain metastasis	Increase	Worsen				[54, 55]
LC	FTO/ IGF2BP1	Overexpression	Poor							[56, 59]
HCC	METTL3/YTHDF1	Overexpression	Poor			Worsen				[58, 121]
HCC	KIAA1429/ IGF2BP1	Overexpression	Poor							[59, 63]
HCC	WTAP	Overexpression	Poor					Prone		[64]
HCC	METTL14	Down-regulation	Better			Weaken	Decrease			[100]
HCC	YTHDF2	Overexpression	Poor	Microvascular invasion		Worsen				[65]
CRC	METTL3	Overexpression	Poor	Lymph node metastasis Liver metastasis Distant metastasis				Prone	Chemotherapy resistance	[67, 68, 70]
CRC	METTL3	Down-regulation	Better		Decrease					[103]
CRC	METTL14	Down-regulation	Better			Weaken	Decrease			[104]
CRC	FTO	Overexpression	Poor		Increase	Worsen				[71]
CRC	YTHDC2	Overexpression	Poor	Lymph node metastasis		Worsen				[74]
CRC	YTHDF1	Overexpression	Poor	Lymph node metastasis Distant metastasis		Worsen				[75]
CRC	IGF2BP2	Overexpression	Poor							[77]
PDAC	METTL3	Overexpression	Poor			Worsen				[78]
GC	METTL3	Overexpression	Poor							[80]
BCA	METTL3	Overexpression	Poor			Worsen	Increase			[82]
BCA	METTL14	Down-regulation	Better			Weaken				[106]
PCA	YTHDF2	Overexpression	Poor				Increase			[85]
RCC	METTL3	Down-regulation	Better		Decrease		Decrease			[107]
RCC	FTO	Down-regulation	Better			Weaken				[108]
RCC	WTAP	Overexpression	Poor		Increase	Worsen				[87]
CSCC	FTO	Overexpression	Poor			Worsen	Increase			[88]
BC	METTL3	Overexpression	Poor							[143]
BC	FTO	Overexpression	Poor				Increase			[94]
EOC	METTL3	Overexpression	Poor			Worsen	Increase			[95]
EOC	ALKBH5	Overexpression	Poor			Worsen	Increase			[96]
EOC	IGF2BP1	Overexpression	Poor							[59]
Melanoma	FTO	Overexpression	Poor			Worsen				[97]
Melanoma	YTHDF1	Down-regulation	Better			Weaken				[110]

*AML* acute myeloid leukemia; *GBM* glioblastoma; *LC* lung cancer; *HCC* hepatocellular carcinoma; *CRC* colorectal cancer; *PDAC* pancreatic cancer; *GC* gastric carcinoma; *BCA* bladder cancer; *PCA* prostate cancer; *RCC* renal cell carcinoma; *CSCC* cervical squamous cell carcinoma; *BC* breast cancer; *EOC* epithelial ovarian cancer

## m6A-related factors in cancer treatment

The balance between methylation and demethylation of m6A at specific RNA transcripts may influence the development of numerous diseases. Thus, regulators or inhibitors of m6A proteins may serve as potential therapeutics for treatment of these diseases (Table 5). M6A inhibitors have been developed for advancing traditional and regenerative medicine, particularly inhibitors of FTO including rhein, R-2HG, IOX3, FB23, MO-I-500, meclofenamic acid and so on [36, 146]. FTO belongs to the family of Fe2+ and 2-oxoglutarate (2OG) dependent AlkB dioxygenases. Meclofenamic acid (MA) was identified as a highly selective inhibitor of FTO [147]. Treatment of GSCs with the ethyl ester form of meclofenamic acid, MA2, could suppress tumorigenesis and prolong the lifespan of GSC-engrafted mice [49]. Afterwards, FB23 and its derivative (FB23–2) display a high selectivity toward FTO. FB23–2 promotes apoptosis and suppresses proliferation of AML cells [148]. Among nonselective inhibitors of FTO, rhein was identified as the first potent FTO inhibitor [149]. As a natural product, rhein competitively binds to the FTO active site and exhibits good inhibitory activity on m6A demethylation [149]. Besides, rhein and MO-I-500 both could decrease tumorigenesis of BC cells [94, 150]. R-2HG could decrease the expression of MYC and alleviates AML and GBM [45]. In addition, knockdown of FTO could enhance the response to AML cells to all-trans retinoic acid (ATRA) treatment and promote ATRA-induced differentiation [44]. In melanoma, the combination of FTO inhibition and anti-PD-1 blockers may reduce resistance to immunotherapy [97]. These collective results indicate that FTO selective or nonselective inhibitors alone or in combination with standard therapeutic agents hold the immense therapeutic potential to cancers, especially those with high FTO expression [151].

**Table 5:** Partial regulators or inhibitors of m6A modifications may provide the potential therapeutic strategies in cancer treatment

Drug	Role	Target	Selective	Biological function	Cancer	Effect/ Mechanism on cancer	Refs
MA /MA2	Inhibitor	FTO	Yes	Stabilize FTO binding for the m6A-containing nucleic acid	GBM	Inhibit GSC growth and self-renewal	[49]
FB23/FB23–2	Inhibitor	FTO	Yes	Directly bind to FTO and inhibit m6A demethylase activity	AML	Suppress proliferation and promote the differentiation/apoptosis	[148]
Rhein	Inhibitor	FTO	No	Binding FTO catalytic domain against ssRNA substrate	AML BC	Prevent or override tyrosine kinase inhibitor resistance Decrease tumor growth	[149] [94]
R-2HG	Inhibitor	FTO	No	Suppress FTO activity and elevate m6A RNA modification	AML	Inhibit proliferation/survival of FTO-high cancer cells	[45]
MO-I-500	Inhibitor	FTO	Yes	Purify FTO demethylase catalyzing demethylation	BC	Inhibit survival of BC cells via decreasing FTO and IRX3 proteins	[150]
SPI1	Regulator	METTL14	No	Negatively regulate METTL14 expression	AML	Inhibit differentiation via targeting MYB and MYC	[42]
CA4	Regulator	WTAP	No	Interact with WTAP and induce WTAP protein degradation	CRC	Inhibits CRC development through WTAP–WT1–TBL1 axis	[73]

*GBM* glioblastoma; *AML* acute myeloid leukemia; *BC* breast cancer; *CRC* colorectal cancer

Previous studies mostly focused on the inhibitors of FTO but other m6A proteins may also be the advantageous target for m6A related cancers. METTL3-depleted cells show a higher sensitivity to anticancer reagents such as gemcitabine, 5-fluorouracil, cisplatin and irradiation in pancreatic cancer [152]. In osteosarcoma, alteration of m6A methylation is associated with acquired chemoresistance [153]. Of mention, 3-deazaadenosine (DAA), a S-adenosylhomocysteine (SAH) hydrolysis inhibitor, has been proven to inhibit METTL3/METTL14 with broad spectrum of effectiveness [154]. Also, Simona et al discovered that small-molecule compounds activate m6A methylation with exceptionally high binding efficiencies to METTL3–14-WTAP. The compounds are experimentally characterized as METTL3–14-WTAP activators that could affect m6A methylation level in HEK293 cells [151]. Rajiv et al proposed a route for further development into potent inhibitors of METTL3. Two series of adenine derivatives were identified and showed good ligand efficiency [155]. Although the pharmacological efficacy of these small-molecule activators or inhibitors of METTL3 has not been reported before, the discovery may open up a new avenue in m6A-targeted pharmacotherapeutics.

Remarkably, several regulators that are upstream of m6A proteins could alter the m6A level via regulating m6A proteins, shedding light on the development of powerful probes and new therapies for cancers. SPI1, a hematopoietic transcription factor, inhibits the development of malignant hematopoietic cells via targeting METTL14 [42]. CA4, a member of the carbonic anhydrases, could interact WTAP and induce WTAP protein degradation, thus suppressing CRC processing through the inhibition of the Wnt signalling pathway [73].

Given that m6A modification has broad physiological functions, its impairment may be a potential novel therapeutic target for the treatment of a wide range of cancers. Specific m6A regulators suitable for clinical trials are thus required. However, it remains a major challenge to identify novel biomarkers and molecular targets to guide therapies in cancers. More selective and efficacious drugs targeting m6A-related factors should be developed and explored.

## Conclusion

**Fig. 3:**
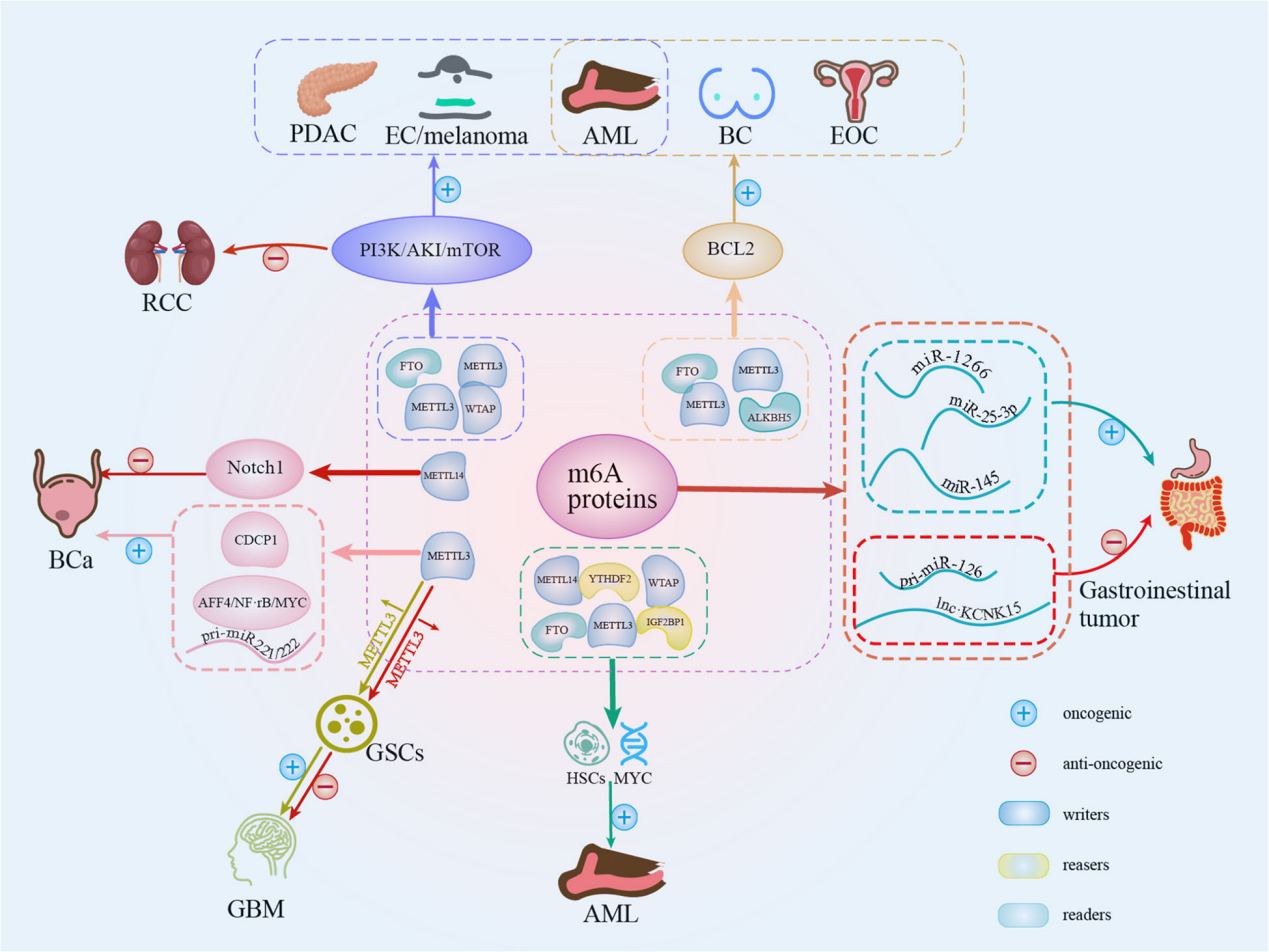
The momentous mechanical pathways of m6A involved in human cancers

As a dominant player in gene expression, m6A is the target of numerous regulatory pathways. The disruption of these mechanisms may result in disease, sometimes with catastrophic consequences. An overview of the mechanical pathways modulated by m6A modification and their implications in human cancers are presented in Fig. 3.

The cross-talk between pathways and co-operation of m6A regulation of gene expression requires considerable study, and at the present, our knowledge is limited. m6A writers, erasers and readers frequently interact with each other, particularly with writers [139]. Sorc et al proposed that METTL3 may regulate WTAP protein homeostasis, and upregulation of WTAP has an oncogenic effect only in the presence of functional METTL3 [156]. The combined outcomes of METTL14, ALKBH5 and YTHDF3 function elevate m6A expression and activity beyond the threshold required to regulate gene expression and activity of critical genes in BC [93]. Additionally, it is a commonly observed phenomena that an m6A-associated protein which participates in different types of cancer are regulated by multiple m6A proteins. For example, there have been contrasting observations suggesting that all the m6A- associated enzymes serve oncogenic roles in AML. Furthermore, an m6A-associated protein in the same type of cancer may regulate different proteins in different individuals. Thus, an extensive amount of effort is required to fully understand the m6A interactome.

Additionally, it remains possible that not all the m6A writers, erasers and readers have been identified. In 2018, Huang et al found that FMR1 and HNRNPC may serve as novel m6A binding proteins [17]. Recently, METTL5 and ZCCHC4 were confirmed to function as exclusive m6A writers of rRNA. Therefore, developing novel m6A detection methods, such as nanopore technology, will assist in the identification of m6A modifiers. For m6A erasers, only two proteins have been identified to date; whereas numerous writers have been discovered. Erasers exhibit diverse biological functions, which may result from their differing tissue distributions and localization. For example, FTO is enriched in the brain and muscle, whereas ALKBH5 is upregulated in the testes [20]. Aberrant expression of FTO or ALKBH5 only results in minor changes in the overall levels of m6A, suggesting that there may be demethylases yet to be discovered. Therefore, identifying novel m6A enzymes may result in the identification of novel regulatory mechanisms.

Importantly, m6A proteins usually serve an oncogenic role in cancer, and the oncogenic role of m6A may be attributed to either promotion of oncogene translation, or initiating the decay of tumor suppressor gene transcripts. However, it is not clear how m6A writers and erasers selectively exert their differing effects, but often still result in the same or similar outcomes, namely the progression of cancer. As an instance, METTL3 may serve dual roles in both GBM and CRC [48, 49, 68, 103]. We proposed the following hypothesis to explain this conflicting phenomena: 1) m6A proteins could function independently of its m6A catalytic activity; 2) Since the fate of m6A-modified mRNAs is also determined by the readers, the difference in the abundance, RNA affinity and cumulative binding of m6A readers may lead to divergent results; 3) The location of the m6A modifications on different regions of the same mRNA transcript may underlie the differing effects; 4) The cross-talk between pathways and co-operation of m6A regulation of gene expression requires considerable study; 5) The conflicting outcomes may also lie in differences in the cancer heterogeneity, cellular context and target specificity of the m6A proteins [37]. However, several questions remain to be answered. How does the methyltransferase family recognize their specific sites and modify them? Does the ncRNA guide the sequence selection?

Remarkably, m6A seldom acts as a tumor-suppressor, excluding METTL14. METTL14 is critical for EBV-associated tumorigenesis through interactions with viral-encoded latent oncoprotein EBNA3C, but in the majority of cases, it serves as a tumor suppressor in several types of cancer, including GBM, HCC, CRC, BCA and EC [99, 104, 139]. These results highlight the impact of m6A modification on the fate of the embedded RNA, and mediation of the RNA function following modification. These key functionally important RNA targets include miRNA, lncRNA and circRNA, amongst others, and are involved in regulating m6A, and may partially explain the mechanism of site selection of m6A. The study of m6A modification of circRNA has recently rose. CircE7 possesses m6A modifications in the cytoplasm, and is translated to produce E7, an oncoprotein, yielding novel insights into how HPV regulates infection and tumorigenesis [157]. M6A modification of circNSUN2 increases export of this circRNA to the cytoplasm, and the export is mediated through the recruitment of YTHDC1, thus enhancing the stability of HMGA2 mRNA to promote progression and metastasis of CRC [30]. Zhang et al demonstrated that m6A modification of the YAP 3′-UTR induces an interaction with miR-382-5p which resulted in the inhibition of YAP, thus impairing the tumorigenic capacity of circRNA_104075 in HCC [158]. Chen et al showed that m6A modifications on human circRNAs inhibit innate immunity through abrogation of immune gene activation, and YTHDF2 is indispensable for suppression of innate immunity [159]. The dual and opposing regulation of m6A modifications and circRNA indicates that interference with the pathway between m6A and immunogenicity of circRNA may be exploited therapeutically.

Methylation of DNA and histone has been the focus of cancer research for several decades, and the DNA methyltransferase inhibitors, azacytidine and decitabine have been approved for cancer therapy in the clinic [160]. As an important RNA epigenetic modification, it remains to be determined how m6A interacts with DNA and histone epigenetics to regulate gene expression, and whether there are potential connections between m6A modifications and other types of RNA modifications. At present, research, and our collective understanding of m6A modifications is still in its infancy.

## Supplementary information

**Additional file 1: Table S1.** The alterations frequency of m6A regulators across 15 cancer types.

**Additional file 2: Figure S1.** The type and percentages of each m6A protein alterations in tumors.

## Data Availability

Not applicable.

## Abbreviations

MYB: Myeloblastosis oncogene
MYC: Myelocytomatosis oncogene
HSPC: Hematopoietic stem and progenitor cells
NANOG: Nanog homeobox
PTEN: Phosphatase and tensin homolog
RARA: Retinoic acid receptor alpha
SOX2: Sex determining region Y box 2
STAT3: Activator of transcription 3
R-2HG: R-2-hydroxyglutarate
SOCS2: Suppressor of cytokine signaling 2
CA4: Carbonic anhydrase IV
eIF: eukaryotic initiation factor
ADAM19: A disintegrin and metallopeptidase domain 19
ASB2: Ankyrin repeat and SOCS box containing 2
BCL2: B cell leukaemia 2
FOXM1: Forkhead box M1
KLF4: Kruppel like factor 4
MALAT1: Metastasis-associated lung adenocarcinoma transcript 1
AFF4: AF4/FMR2 family member 4
PPAR: Peroxisome proliferator-activator
