# Supplementary material for: Mechanism of RNA modification N6-methyladenosine in human cancer
Source: Mol Cancer. 2020 Jun 8;19:104. doi: 10.1186/s12943-020-01216-3 (PMC7278081; doi:10.1186/s12943-020-01216-3)
Supplement: Supplementary file 1 — Additional file 1: Table S1. The alterations frequency of m6A regulators across 15 cancer types. [file 12943_2020_1216_MOESM1_ESM.docx]

**Table S1. The alterations frequency of m6A regulators across 15 cancer types.**

|  | **AML** | **GBM** | **LC** | **HCC** | **GC** | **PDAC** | **CRC** | **BCA** | **PCA** | **RCC** | **BC** | **EC** | **EOC** | **CSSC** | melanoma |
| --- | --- | --- | --- | --- | --- | --- | --- | --- | --- | --- | --- | --- | --- | --- | --- |
| **METTL3** | 0.040 | 0.028 | 0.050 | 0.040 | 0.050 | 0.080 | 0.050 | 0.080 | 0.040 | 0.040 | 0.050 | 0.080 | 0.030 | 0.070 | 0.060 |
| **METTL14** | 0.060 | 0.060 | 0.050 | 0.030 | 0.050 | 0.050 | 0.050 | 0.050 | 0.050 | 0.050 | 0.050 | 0.080 | 0.050 | 0.060 | 0.050 |
| **WTAP** | 0.050 | 0.030 | 0.050 | 0.050 | 0.060 | 0.050 | 0.070 | 0.060 | 0.070 | 0.060 | 0.060 | 0.110 | 0.060 | 0.060 | 0.070 |
| **KIAA1429** | 0.050 | 0.070 | 0.080 | 0.060 | 0.090 | 0.070 | 0.100 | 0.100 | 0.050 | 0.030 | 0.060 | 0.140 | 0.060 | 0.080 | 0.130 |
| **RBM15** | 0.030 | 0.030 | 0.050 | 0.050 | 0.070 | 0.050 | 0.070 | 0.080 | 0.060 | 0.050 | 0.050 | 0.090 | 0.060 | 0.070 | 0.050 |
| **RBM15B** | 0.040 | 0.080 | 0.050 | 0.050 | 0.080 | 0.050 | 0.070 | 0.060 | 0.060 | 0.040 | 0.050 | 0.100 | 0.060 | 0.030 | 0.080 |
| **HNRNPA2B1** | 0.050 | 0.050 | 0.050 | 0.050 | 0.060 | 0.050 | 0.060 | 0.060 | 0.040 | 0.050 | 0.050 | 0.090 | 0.040 | 0.025 | 0.070 |
| **HNRNPC** | 0.050 | 0.040 | 0.070 | 0.050 | 0.050 | 0.040 | 0.050 | 0.070 | 0.050 | 0.050 | 0.050 | 0.080 | 0.050 | 0.050 | 0.060 |
| **ZC3H13** | 0.070 | 0.060 | 0.070 | 0.060 | 0.120 | 0.050 | 0.100 | 0.090 | 0.050 | 0.060 | 0.070 | 0.160 | 0.070 | 0.100 | 0.120 |
| **FTO** | 0.060 | 0.060 | 0.080 | 0.050 | 0.070 | 0.060 | 0.060 | 0.070 | 0.050 | 0.060 | 0.060 | 0.080 | 0.060 | 0.060 | 0.040 |
| **ALKBH5** | 0.060 | 0.060 | 0.050 | 0.050 | 0.050 | 0.050 | 0.050 | 0.060 | 0.060 | 0.050 | 0.050 | 0.070 | 0.070 | 0.070 | 0.060 |
| **YTHDC1** | 0.040 | 0.050 | 0.060 | 0.050 | 0.070 | 0.050 | 0.080 | 0.070 | 0.050 | 0.070 | 0.050 | 0.120 | 0.040 | 0.050 | 0.090 |
| **YTHDC2** | 0.080 | 0.060 | 0.070 | 0.050 | 0.040 | 0.050 | 0.090 | 0.070 | 0.040 | 0.070 | 0.050 | 0.140 | 0.040 | 0.080 | 0.100 |
| **YTHDF1** | 0.024 | 0.028 | 0.050 | 0.050 | 0.050 | 0.070 | 0.030 | 0.060 | 0.060 | 0.050 | 0.050 | 0.080 | 0.060 | 0.070 | 0.060 |
| **YTHDF2** | 0.050 | 0.060 | 0.050 | 0.050 | 0.070 | 0.050 | 0.060 | 0.070 | 0.060 | 0.060 | 0.040 | 0.100 | 0.050 | 0.070 | 0.040 |
| **YTHDF3** | 0.050 | 0.080 | 0.060 | 0.060 | 0.070 | 0.050 | 0.060 | 0.050 | 0.060 | 0.060 | 0.060 | 0.100 | 0.070 | 0.050 | 0.070 |
| **IGF2BP1** | 0.000 | 0.080 | 0.028 | 0.003 | 0.070 | 0.050 | 0.090 | 0.040 | 0.040 | 0.060 | 0.040 | 0.110 | 0.030 | 0.040 | 0.150 |
| **IGF2BP2** | 0.040 | 0.060 | 0.070 | 0.050 | 0.080 | 0.050 | 0.050 | 0.030 | 0.050 | 0.050 | 0.090 | 0.070 | 0.040 | 0.070 | 0.060 |
| **IGF2BP3** | 0.024 | 0.060 | 0.090 | 0.011 | 0.030 | 0.040 | 0.023 | 0.017 | 0.027 | 0.060 | 0.070 | 0.070 | 0.010 | 0.050 | 0.070 |

The results in our analysis were based upon cBioPortal database (https://www.cbioportal.org/). We totally analyzed 15 different TCGA projects (TCGA, PanCancer Atlas), each project represents a specific cancer type, including AML, acute myeloid leukemia; GBM, glioblastoma multiforme; LC, lung squamous cell carcinoma; HCC, liver hepatocellular carcinoma; GC, stomach adenocarcinoma; PDAC, pancreatic adenocarcinoma; CRC, colorectal adenocarcinoma; BCA, bladder urothelial carcinoma; PCA, prostate adenocarcinoma; RCC, kidney renal clear cell carcinoma; BC, Breast Invasive Carcinoma; EC, uterine corpus endometrial carcinoma; EOC, ovarian serous cystadenocarcinoma; CSSC, cervical squamous cell carcinoma, melanoma, Skin Cutaneous Melanoma
