# Supplementary figures and images for: Mechanism of RNA modification N6-methyladenosine in human cancer
Source: Mol Cancer. 2020 Jun 8;19:104. doi: 10.1186/s12943-020-01216-3 (PMC7278081; doi:10.1186/s12943-020-01216-3)

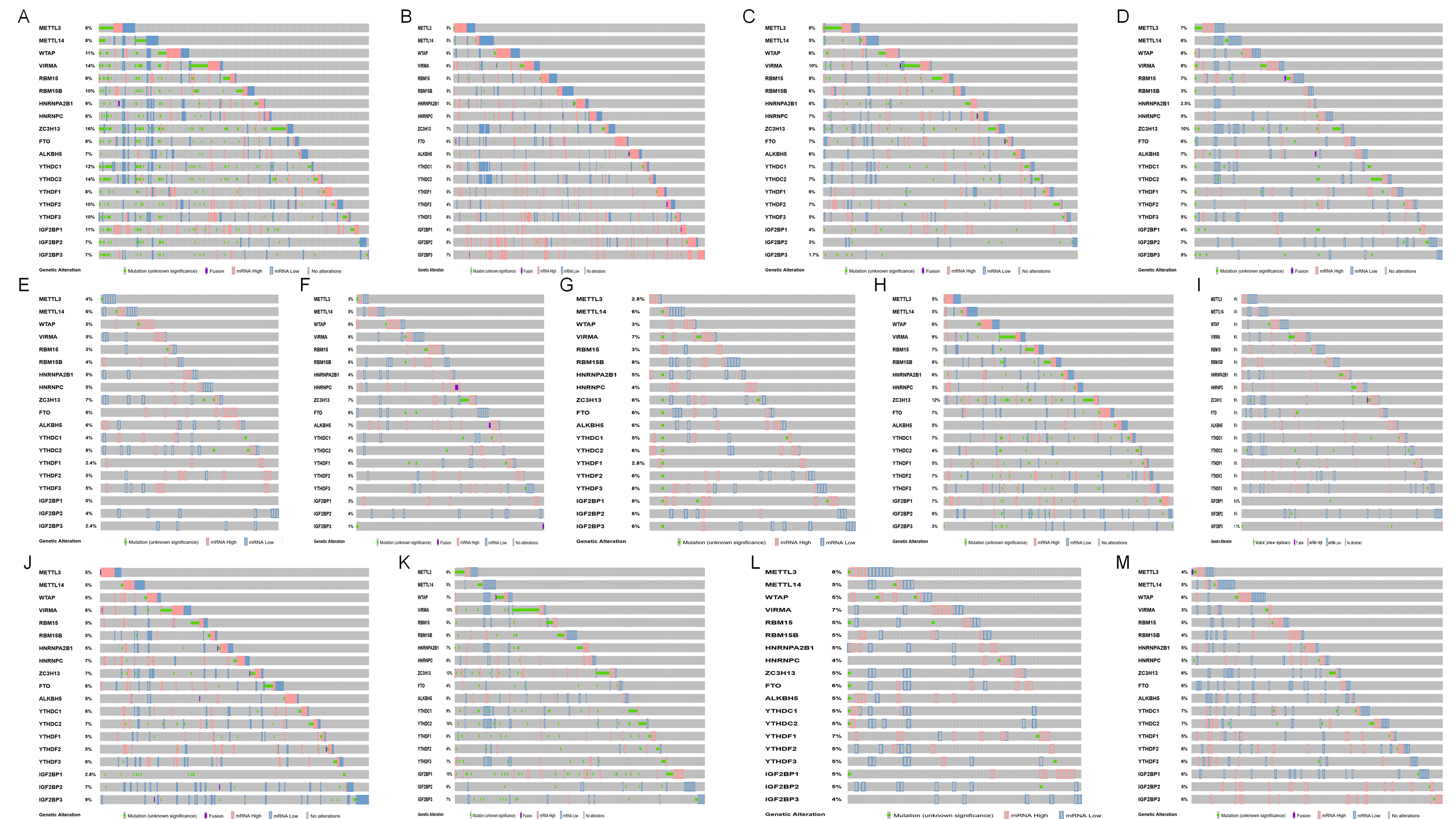

Supplement: Supplementary file 2 — Additional file 2: Figure S1. The type and percentages of each m6A protein alterations in tumors. [file 12943_2020_1216_MOESM2_ESM.tif]
